# Cooperativity in microbial biotechnology: synthetic consortia as emerging metabolic engineering strategy for sustainable bioproduction

**DOI:** 10.3389/fbioe.2026.1820441

**Published:** 2026-06-10

**Authors:** Fatma Gizem Avci, Volker F. Wendisch

**Affiliations:** 1 Genetics of Prokaryotes, Faculty of Biology and Center for Biotechnology (CeBiTec), Bielefeld University, Bielefeld, Germany; 2 Department of Bioengineering, Faculty of Engineering and Natural Sciences, Üsküdar University, Istanbul, Türkiye

**Keywords:** bioproduction, *Corynebacterium glutamicum*, *Escherichia coli*, metabolic engineering, *Pseudomonas putida*, sustainability, synthetic consortia

## Abstract

Microbial cell factories play an important role in the sustainable production of chemicals used in several industries, including pharmaceutical, food, polymer, and energy. Biosynthesis of these desired chemicals typically occurs through complex or extended metabolic pathways *via* multiple enzymatic steps. However, introducing multiple heterologous genes into a single microbial strain often creates a significant metabolic burden, making the efficient production of target chemicals challenging. To overcome the limitations of monocultures, microbial consortia can be engineered to divide complex catabolic or biosynthetic tasks among different microbial partners. In contrast to monocultures, which often suffer from metabolic burden, pathway interference, and limited tolerance to toxic intermediates, consortia-based systems may benefit from a modular division of labor. This approach enables efficient utilization of metabolic resources, enhanced pathway flux, and improved system robustness. This review focuses on the bioproduction of various target compounds by synthetic microbial consortia containing *Corynebacterium glutamicum*, *Escherichia coli*, or *Pseudomonas putida* at least as one partner. Where relevant, a particular focus will be laid on cooperativity in mutualistic consortia.

## 1 Introduction

Microbiomes, microbiota, heterogenous microbial populations, co-cultures, natural and synthetic microbial consortia are attracting a lot of attention in various research areas ranging from medicine (e.g., the human gut microbiome upon drug treatment (Garcia-Santamarina et al., 2024)), ecology (e.g., the pampa (Granada et al., 2019)) or arctic fur seal microbiomes (Grosser et al., 2019)), fermented foods (e.g., the kefir microbiome (Chong et al., 2023)) or the synthetic microbial consortia of biotechnological relevance that are covered in this review.

In natural ecosystems, microorganisms predominantly exist within highly complex and dynamic consortia, where individual species fulfill distinct roles shaped by population density, environmental conditions, and resource availability. Through extensive interactions among their members and the environment, they perform highly complex functions, such as metabolic transformations, that cannot be achieved by a single organism alone (García-Jiménez et al., 2021; Matuszýnska et al., 2024). Alongside the growing recognition of the roles of natural microbial consortia, advances in molecular biology have significantly improved the precision and versatility of genetic tools, driving an increasing interest in engineering complex natural communities and in the design of synthetic microbial consortia (Mittermeier et al., 2023; Matuszýnska et al., 2024).

Engineering synthetic microbial consortia offers, in an abstract bottom-up perspective, to identify the underlying principles of coexistence and collaboration of natural consortia. With biotechnological applications in mind, engineering synthetic microbial consortia has created new opportunities in synthetic biology by enhancing system complexity and functional potential, and has provided several advantages over monocultures. For example, in a division of labor approach, metabolic pathways can be partitioned among different chassis organisms, minimizing interference between distinct functions. Co-cultures can be composed of diverse strains, creating optimal catalytic environments for enzymes originating from different sources. Cofactors and energy resources, such as NADH and ATP, can be more effectively balanced across specialized microorganisms. Pathway regulation can be achieved flexibly by adjusting strain ratios rather than relying on complex genetic modifications. Moreover, intercellular interactions promote dynamic equilibrium within co-culture systems, enhancing their adaptability and stability under fluctuating environmental conditions (Pan et al., 2023).

A systematic understanding of consortia design principles is important to maximize the desired functions. Metabolic pathway partitioning aims to increase efficiency by distributing complex metabolic processes among different specialized strains, rather than assigning the metabolic load to a single microorganism. It is most advantageous when single hosts experience metabolic burden, toxic intermediate accumulation, or poor cofactor balance. By splitting the pathway among different strains in a synthetic consortium, the overall metabolic burden is divided, thereby alleviating stress and improving system robustness (Tsoi et al., 2018; Roell et al., 2019). In some cases, intermediates may be exchanged between multiple populations. However, restricted transport across cell membranes and dilution in the extracellular environment can lower metabolic efficiency by decreasing the effective concentrations of substrates or enzymes. Metabolic pathways can be engineered to reduce intermediate loss to overcome this limitation (Tsoi et al., 2018). A critical aspect of consortium design is the selection of pathway splitting points (nodes). These nodes are strategically chosen along biosynthetic routes to optimize flux distribution and overall productivity, avoid the buildup of toxic intermediates, maintain balanced metabolic flux, and support the effective exchange of metabolites between different strains. Nodes with stable intermediate pools, well-characterized transport mechanisms, and reasonable thermodynamic driving force are suitable for splitting (Bekiaris and Klamt, 2021; Lin et al., 2024).

In general, six types of interaction mechanisms are observed within microbial consortia: neutralism, amensalism, predation, competition, commensalism, and mutualism. Mutualism is advantageous when stable, long-term coexistence between partners is needed. Commensalism is appropriate in cases where one microorganism mainly supplies a precursor metabolite that is utilized by another. Neutralism, on the other hand, applies when partners have minimal impact on each other’s growth but still collaborate through division of substrate use (Jiang et al., 2023).

Before we describe the manifold biotechnological applications of microbial consortia, we will provide definitions that we deem helpful with regard to this review. Ecological interactions between two community members are generally classified based on the positive or negative fitness consequences they have for the interacting partners. Since we did not find synthetic microbial consortia with applications in biotechnology that include negative fitness consequences by the consortial partner (Jiang et al., 2023), we only cover synthetic microbial consortia with neutral or beneficial fitness consequences.

**FIGURE 1:**
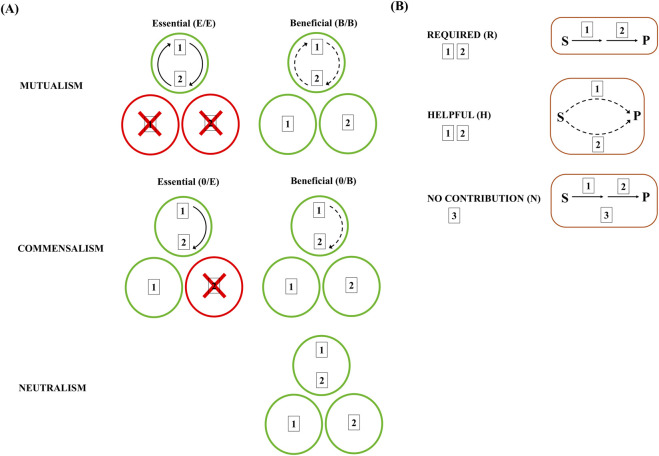
Schematic representation of fitness consequences **(A)** in binary microbial consortia and production schemes **(B)** by microbial consortia. **(A)** Microbial partners may coexist either without (0), with essential (E, no survival without the partner, solid lines), or with beneficial (B, better survival with the partner, hatched lines) positive fitness consequences. Mutualism is characterized by interdependent benefits of both partners, while in commensalism, only one partner benefits. In addition to consortia, viable (green circle) and non-viable (red cross and red circle) monocultures of the respective partners are also shown. **(B)** Microbial partners may either contribute a required (R) or helpful (H) reaction to a production route or do not contribute to a production route (N).

Specifically, we distinguish positive fitness consequences as essential (E, no survival without the partner), beneficial (B, better survival with the partner), or neutral (0, no advantage with the partner) (Figure 1A). Mutualism in binary consortia is characterized by interdependencies of both partners and can either be obligate (E/E, neither partner can survive without the other) or facultative (B/B, both partners strive better with the other, but do not require the partner for survival). Commensalism in binary consortia describes interactions where positive fitness consequences affect only one partner (either 0/E, if the dependent partner requires the other for survival, or 0/B, if the dependent partner strives better with the other, but their interaction is not required for survival). Neutralism (0/0) in binary consortia occurs when both partners coexist, but their fitness is unaffected. Ternary, quaternary, and higher-order consortia can be categorized by deconvolution to binary sub-interactions.

Production by microbial consortia may be categorized with respect to the sequence of reactions in cascades (without considering focus on fitness consequences on growth or survival) (Figure 1B). The microbial partner is either not contributing to the reaction cascade (N) or is active as a catalyst, exerting a reaction that is either helpful (H) or required (R) for the cascade to run to completion. Consortia may collaborate in production in cascades that are linear, U-shaped, convergent, or with more complex layouts. Reaction cascades catalyzed by synthetic microbial consortia that involve three or more partners can be categorized by deconvolution to binary sub-interactions. In this review, we do not consider substrate competition if one carbon source is used by more than one partner, since biotechnological production can be operated such that the joint substrate is not a limiting factor, as it is either added in surplus in batch cultivations or added continuously in fed-batch or continuous cultivations.

The bacteria *Escherichia coli*, *Corynebacterium glutamicum*, and *Pseudomonas putida* have in common playing important roles in industrial biotechnology and have been used in synthetic microbial consortia not only for conceptual studies, but specifically also for fermentative production of valuable compounds. Therefore, this review focuses on bioproduction by synthetic microbial consortia with at least one of the partners being *E. coli*, *C. glutamicum*, or *P. putida*. The synthetic microbial consortia described involve, *i.a.*, yeasts, bacilli, clostridia, and cyanobacteria as further partner(s).

## 2 Production by microbial consortia with division of labor

In natural environments, microorganisms form complex communities that cooperate through cellular division of labor, enabling functional specialization, efficient resource utilization, and enhanced competitive fitness (Giri et al., 2019). In contrast, biotechnology predominantly employs monocultures, engineering organisms as tightly controlled whole-cell biocatalysts to perform complex bioprocesses with a single step. However, genetically introduced functions often compromise host performance through unintended pathway interference, increased metabolic burden that reduces growth and productivity, and accumulation of toxic intermediates or products (Tsoi et al., 2018; Ceballos Rodriguez-Conde et al., 2025). To address these challenges, the division of labor between two or more partners in specifically designed microbial consortia represents an alternative. This strategy can be applied as (i) pathway splitting*,* where sequential biosynthetic steps are allocated to different strains (ii) substrate partitioning, where carbon sources (e.g., mixed sugars) are divided among partners; and (iii) dependency-based cooperation (mutualistic dependency), where growth or survival depends on cross-feeding (Roell et al., 2019). These frameworks guide the selection of representative examples and the design strategies. In the following, we will highlight illustrative examples of pathway splitting and substrate partitioning with different consortia design patterns, including linear, U-shaped, convergent, Y-shaped, and tooth squeege-shaped, among many applications listed in Tables 1–3.

**TABLE 1:** Metabolically engineered synthetic consortia with *C. glutamicum* as one partner at least. Growth interaction either does not exist (0), has essential (E, no survival without the partner) or beneficial (B, better survival with the partner) positive fitness consequences. The contribution of a microbial partner to product interaction may be required (R), helpful (H), or not existing (N).

Microorganism	Growth interaction	Production interaction	Product	References
BINARY CONSORTIA				
*C. glutamicum–P. putida*	E/E	N/R	γ-glutamyl-isopropylamide (GIPA) or L-theanine	Benninghaus et al. (2024)
*C. glutamicum–E. coli*	0/E E/E	R/N R/N	L-lysine L-lysine, cadaverine, or L-pipecolic acid	Sgobba et al. (2018)
*C. glutamicum–E. coli*	E/E	R/N	L-lysine	Vortmann et al. (2021)
*C. glutamicum–Synechococcus elongatus*	E/0	R/R	*cis,cis-*muconate	Ma et al. (2026)
*C. glutamicum–C. glutamicum*	0/0	R/R	Tyrosol	Junker et al. (2025)
*C. glutamicum–Bacillus subtilis*	0/B	H/R	Fengycin	Gao et al. (2023)
*C. glutamicum–E. coli*	0/B	H/R	Violacein	Gwon et al. (2023)
*C. glutamicum–E. coli*	0/B	H/R	Cadaverine	Liu et al. (2022b)
*C. glutamicum–Paenibacillus polymyxa*	0/B	H/R	Polymyxin	Sun et al. (2024)
*C. glutamicum–C. glutamicum*	0/0	R/R	Resveratrol	Mutz et al. (2023)
*C. glutamicum–Bacillus amyloliquefaciens*	E/B	H/R	Lipopeptide (Fengycin, surfactin, iturin A)	Chen et al. (2022)
TERNARY CONSORTIA				
*C. glutamicum–B. amyloliquefaciens–P. pastoris*	E/B/0	H/R/N	Lipopeptide (Fengycin, surfactin, iturin A)	Chen et al. (2022)
*C. glutamicum–B. subtilis–Yarrowia lipolytica*	0/B/0	H/R/H	Fengycin	Wei et al. (2024)
*C. glutamicum–B. amyloliquefaciens–Y. lipolytica*	E/B/E	H/R/H	Lipopeptide (Fengycin, surfactin, iturin A)	Zhang et al. (2024)
*C. glutamicum–B. subtilis–Y. lipolytica*	0/B/0	H/R/H	Fengycin	Duan et al. (2025)
*C. glutamicum–C. glutamicum–B. amyloliquefaciens*	0/0/B	H/H/R	Lipopeptide (Fengycin, surfactin, iturin A)	Sun et al. (2022)
*C. glutamicum–B. amyloliquefaciens–Y. lipolytica*	E/B/E	H/R/H	Lipopeptide (Fengycin, surfactin, iturin A)	He et al. (2024)
QUATERNARY CONSORTIA				
*C. glutamicum–C. glutamicum–B. amyloliquefaciens–Y. lipolytica*	E/E/B/E	H/H/R/H	Lipopeptide (Fengycin, surfactin, iturin A)	He et al. (2024)
*C. glutamicum–C. glutamicum–B. amyloliquefaciens–P. pastoris*	E/E/B/0	H/H/R/N	Lipopeptide (Fengycin, surfactin, iturin A)	Sun et al. (2022)
*C. glutamicum–C. glutamicum–C. glutamicum–C. glutamicum*	0/0/0/0	H/H/H/H	Riboflavin	Pérez-García et al. (2021)

**TABLE 2:** Metabolically engineered synthetic consortia with *P. putida* as one partner (s. Table 1 for explanation of abbreviations and the *C. glutamicum–P. putida* consortia).

Microorganism	Growth interaction	Production interaction	Product	References
BINARY CONSORTIA				
*S. elongatus–P. putida*	0/E	N/R	2,5-furandicarboxylic acid	Lin et al. (2020)
*E. coli–P. putida*	E/E	R/R	D-*p*-hydroxyphenylglycine	Liu et al. (2022e)
*Saccharomyces cerevisiae–P. putida*	E/0	H/R	Medium chain length polyhydroxyalkanoates (mcl-PHA)	Wei et al. (2023)
*E. coli–P. putida*	B/0	H/R	mcl-PHA	Zhu et al. (2022)
*E. coli–P. putida*	B/0	H/R	mcl-PHA	Qin et al. (2022)
*Bacillus coagulans–P. putida*	B/0	R/N	Lactate	Zou et al. (2021)
*P. polymyxa–P. putida*	B/0	R/N	2,3-butanediol	Rama et al. (2025)
*S. elongatus–P. putida*	0/E	N/R	PHA	Kratzl et al. (2023)
*S. elongatus–P. putida*	0/E	N/R	PHA	Hobmeier et al. (2020)
*S. elongatus–P. putida*	0/E	N/R	Indigoidine	Zhao et al. (2022)

**TABLE 3:** Metabolically engineered synthetic consortia with *E. coli* as one partner at least (s. Table 1 for explanation of abbreviations and the *C. glutamicum–E. coli* consortia, s. Table 2 for *P. putida–E. coli* consortia).

Microorganism	Growth interaction	Production interaction	Product	References
BINARY CONSORTIA				
*S. cerevisiae–E. coli*	E/B	R/H	Naringenin	Zhang et al. (2017)
*Eubacterium limosum–E. coli*	B/E	N/R	3-hydroxypropionic acid or itaconic acid	Cha et al. (2021)
*Meyerozyma guilliermondii–E. coli*	B/B	R/H	2-phenylethanol	Yan et al. (2022)
*S. cerevisiae–E. coli*	E/B	R/R	Oxygenated taxanes	Zhou et al. (2015)
*E. coli–E. coli*	E/E	R/N	Phenylalanine	Kawai et al. (2022)
*E. coli–E. coli*	E/E	R/R	Salidroside	Liu et al. (2018)
*E. coli–E. coli*	E/E	R/R	Violacein	Schick et al. (2024)
*E. coli–E. coli*	0/0	R/R	Kaempferide	Qiu et al. (2022)
*E. coli–E. coli*	0/0	N/R	n-butanol	Saini et al. (2017)
*B. subtilis–E. coli*	B/E	N/R	Coniferol or chavicol	Chacόn et al. (2024)
*Methylococcus capsulatus–E. coli*	0/E	N/R	Mevalonate	Lee et al. (2021)
*E. coli–E. coli*	0/0	R/R	Pinene	Niu et al. (2018)
*E. coli–E. coli*	0/0	R/R	Multi-methyl-branched esters	Bracalente et al. (2022)
*E. coli–E. coli*	0/B	H/R	Indigo	Chen et al. (2021)
*E. coli–E. coli*	0/0	R/R	Bisdemethoxycurcumin	Fang et al. (2018)
*E. coli–E. coli*	0/B	H/R	4-hydroxystyrene	Gargatte et al. (2021)
*E. coli–E. coli*	0/0	R/R	Hydroxytyrosol	Gong et al. (2023)
*E. coli–E. coli*	0/0	R/R	Afzelechtin or catechin	Jones et al. (2016)
*S. cerevisiae–E. coli*	0/0	R/R	Hydroxytyrosol	Liu et al. (2022d)
*E. coli–E. coli*	0/B	H/R	Cadaverine	Wang et al. (2018)
*E. coli–E. coli*	0/0	R/R	Hesperetin	Liu et al. (2022a)
*E. coli–E. coli*	B/0	R/H	D-pantothenic acid	Qin et al. (2025a)
*E. coli–E. coli*	0/0	R/R	1,6-hexanediol or 1,6-hexamethylenediamine	Qin et al. (2025b)
*E. coli–E. coli*	0/0	R/R	Vanilin	Qiu et al. (2025a)
*E. coli–E. coli*	0/0	R/R	n-butanol	Saini et al. (2016)
*E. coli–E. coli*	0/0	R/R	Apigetrin	Thuan et al. (2018a)
*E. coli–E. coli*	0/0	R/R	Resveratroloside or polydatin	Thuan et al. (2018b)
*E. coli–E. coli*	0/0	R/R	Eriodictyol	Thuan et al. (2022)
*E. coli–E. coli*	0/0	R/R	Genkwanin	Thuan et al. (2023)
*P. pastoris–E. coli*	0/0	R/R	Stylopine	Urui et al. (2021)
*E. coli–E. coli*	0/0	R/R	Glutarate	Wang et al. (2019b)
*E. coli–E. coli*	0/0	R/R	Acacetin	Wang et al. (2020d)
*E. coli–E. coli*	0/0	R/R	Sakuranetin	Wang et al. (2020b)
*E. coli–E. coli*	0/B	H/R	Tryptamine	Wang et al. (2020c)
*E. coli–Candida glycerinogenes*	0/B	H/R	Caffeic acid	Wang et al. (2024)
*E. coli–E. coli*	0/0	R/R	3-hydroxyphloretin	Won et al. (2025)
*E. coli–E. coli*	0/0	R/R	Pterostilbene	Yan et al. (2023)
*S. cerevisiae–E. coli*	0/0	R/R	Resveratrol	Yuan et al. (2020)
*E. coli–E. coli*	0/0	R/R	1,3-propanediol	Yun et al. (2021)
*E. coli–E. coli*	0/B	H/R	3-amino-benzoic acid	Zhang and Stephanopoulos (2016)
*E. coli–E. coli*	0/B	H/R	*cis,cis*-muconic acid	Zhang et al. (2015)
*E. coli–E. coli*	0/0	R/H	Gallic acid	Wang et al. (2025)
*Elizabethkingia meningoseptica–E. coli*	0/0	H/H	Vitamin K2	Yang et al. (2022)
*S. cerevisiae–E. coli*	0/0	H/H	Ethanol	Wang et al. (2019a)
*E. coli–E. coli*	0/0	H/H	Pyruvate	Maleki et al. (2018)
*E. coli–E. coli*	0/0	N/N	Lactate and succinate	Flores et al. (2020)
*Chlamydomonas reinhardtii–E. coli*	0/E	N/R	Lycopene	Kang et al. (2025)
*S. elongatus–E. coli*	0/E	N/R	3-hydroxypropionic acid	Zhang et al. (2020)
*S. cerevisiae–E. coli*	0/0	R/R	Strigolactone	Wu et al. (2021)
*E. coli–E. coli*	0/0	R/R	Pyranoanthocyanins	Akdemir et al. (2019)
*E. coli–E. coli*	0/E	N/R	Isopropanol	Honjo et al. (2019)
*B. subtilis–E. coli*	0/0	N/N	Lipase and D-psicose	Zhang et al. (2021)
*E. coli–E. coli*	0/B	H/R	4–hydroxybenzoic acid	Zhao et al. (2025)
*E. coli–E. coli*	0/0	H/H	Butanol	Zhao et al. (2019)
*Komagataeibacter xylinus–E. coli*	0/0	R/R	Colored bacterial cellulose	Zhou et al. (2025)
*E. coli–S. cerevisiae*	0/0	R/R	Dihydro-β-ionone	Ke et al. (2026)
*E. coli–E. coli* *E. coli–E. coli* *E. coli–E. coli*	0/0 0/0 E/0	R/R R/R R/R	Flavonoids or flavonoid glycosides	Qiu et al. (2025b)
TERNARY CONSORTIA				
*E. coli–E. coli–E. coli*	0/0/0	R/R/R	Acacetin	Wang et al. (2020d)
*E. coli–E. coli–E. coli* *E. coli–E. coli–E. coli*	0/0/0 B/B/B	R/R/R R/R/R	Flavonoids or flavonoid glycosides	Qiu et al. (2025b)
*E. coli–E. coli–E. coli*	0/0/0	R/R/R	Eugenol or hydroxychavicol	Brooks et al. (2023)
*E. coli–E. coli–E. coli*	0/0/0	R/R/R	Protocatechuic acid and hydroquinone	Guo et al. (2020)
*Y. lipolytica–E. coli–E. coli*	0/0/0	R/H/R	α,ω-diamines	Kim et al. (2024)
*E. coli–E. coli–E. coli*	E/0/0	R/R/R	Chlorogenic acid	Li et al. (2021)
*E. coli–E. coli–E. coli*	0/0/0	R/R/R	Genistein	Liu et al. (2022c)
*E. coli–E. coli–E. coli*	0/0/0	R/R/R	Rosmarinic acid	Li et al. (2019)
*E. coli–E. coli–E. coli*	0/0/0	R/R/R	α,ω-dicarboxylic acids	Wang et al. (2020a)
*Clostridium acetobutylicum–Clostridium acetobutylicum–E. coli*	B/B/B	R/R/R	Butyl butyrate	Lu et al. (2023)
QUATERNARY CONSORTIA				
*C. acetobutylicum–C. tyrobutyricum–T. asperellum–E.coli*	E/E/0/E	R/R/N/R	Butyl butyrate	Lu et al. (2023)
*Thermosynechococcus elongatus–S.elongatus–E. coli–E. coli*	0/0/E/E	N/N/R/R	Ethylene and isoprene	Cui et al. (2022)
*E. coli–E. coli–E. coli–E. coli*	0/0/0/0	R/R/R/R	Anthocyanins	Jones et al. (2017)

**FIGURE 2:**
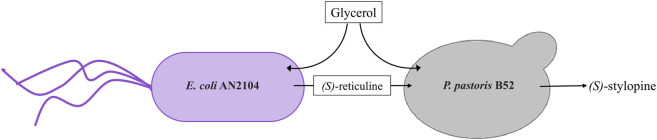
Linear production (type R/R) of (*S*)-stylopine requiring both partners of the binary *E. coli*–*P. pastoris* consortium (Urui et al., 2021). The growth of both partners with glycerol as a carbon and energy source is independent (0/0), whereas both strains contribute required reactions to (*S*)-stylopine production. *E. coli* strain AN2104 is required to produce and secrete intermediate (*S*)-reticuline, which *P. pastoris* strain B52 imports and converts to (*S*)-stylopine, which is then secreted (Table 2).

Several benzylisoquinoline alkaloids, including the anti-inflammatory stylopine, can be biosynthesized from (*S*)-reticuline as the key intermediate. Stylopine production has been realized in a binary consortium. *E. coli* and *Pichia pastoris* were engineered for division of labor regarding the *de novo* biosynthesis of this compound in a linear cascade (Figure 2). *E. coli* (upstream module) converted a simple carbon source, glycerol, into (*S*)-reticuline, which was subsequently transformed into stylopine by the downstream *P. pastoris* module. Further analysis of the initial inoculation ratio revealed that increasing the proportion of *E. coli* relative to *P. pastoris* enhanced stylopine production (Urui et al., 2021). Changing the stoichiometry between the two strains by different inoculation ratios was possible, since the consortium did not include growth (inter)dependencies.

**FIGURE 3:**
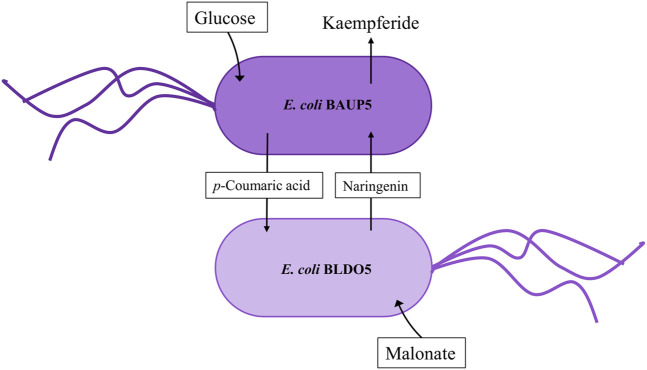
U-shaped production (type R/R) of kaempferide requiring both *E. coli* partners of the binary consortium (Qiu et al., 2022). While survival of both partners does not depend on the other (0/0), both are required for kaempferide production (R/R). Strain BAUP5 is required to produce and secrete intermediate *p*-coumaroyl-CoA and to convert naringenin to kaempferide, while strain BLDO5 is required to convert *p*-coumaroyl-CoA to naringenin, which is secreted (Table 3).

The synthesis of kaempferide, an *O*-methylated flavonol from *Kaempferia galanga*, was performed in monoculture and consortia (Qiu et al., 2022). Due to its complexity, the kaempferide biosynthesis pathway was divided into upstream (from glucose to *p*-coumaric acid), midstream (from *p*-coumaric acid to naringenin), and downstream modules (from naringenin to kaempferide). In monoculture, 18.8 ± 1.0 mg/L kaempferide was the highest titer obtained, although a higher amount of *p*-coumaric acid (164.3 ± 7.1 mg/L) was produced. This was explained by a significant metabolic burden due to an imbalance between the modules within the long kaempferide biosynthetic pathway. To address this limitation, binary *E. coli–E. coli* consortia were engineered using naringenin or *p*-coumaric acid as the metabolic nodes with a linear division pattern. In the first consortium, one strain produced naringenin, while the other converted it to kaempferide. In this co-culture system, 21.7 ± 1.1 mg/L kaempferide was obtained by testing different inoculation ratios. To reduce the metabolic burden on the first strain, the midstream module was transferred to the second strain. By using the *p*-coumaric acid as the node, the second consortium produced 48.2 ± 2.0 mg/L kaempferide, which was 1.2-fold higher than that produced by the first consortium. Because the midstream module that converts *p*-coumaric acid to naringenin demands more cellular resources than the other modules, it may lead to reduced overall performance. Therefore, a three-strain consortium was constructed by separating the midstream module from the downstream module. After tuning the inoculation ratios of the three strains, 77.4 ± 1.4 mg/L kaempferide was produced. Consequently, a “U-shaped” co-culture was designed, in which *p*-coumaric acid and kaempferide biosynthesis were hosted in one strain and naringenin biosynthesis in the other (Figure 3). This co-culture system was robust during the fermentation process and enhanced the production of kaempferide (116.0 ± 3.9 mg/L). This highlights the importance of metabolic node selection and module division in co-culture design.

**FIGURE 4:**
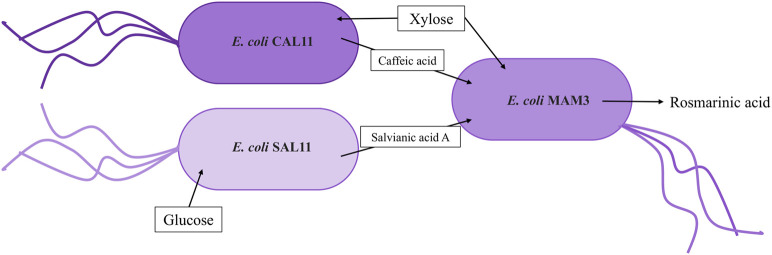
Convergent production of rosmarinic acid (type R/R/R) by a ternary *E. coli* consortium (Li et al., 2019). Three strains can grow independently (0/0/0). Strains CAL11 and MAM3 utilize xylose, while strain SAL11 grows with glucose. Strain MAM3 imports salvianic acid and caffeic acid to combine them to rosmarinic acid, which is secreted. Rosmarinic acid production requires contributions by each of the three strains (Table 3).

The nonlinear rosmarinic acid biosynthetic pathway was constructed in a converging ternary consortium as a representative example of modular co-culture engineering with the successful balancing of *E. coli* strains (Li et al., 2019). In its converging biosynthetic pathway, rosmarinic acid was formed by condensation of two precursors, caffeic acid and salvianic acid A. First, a binary co-culture in which one strain was solely responsible for the formation of precursor caffeic acid, while the downstream strain produced both the salvianic acid A and rosmarinic acid was constructed. Competition for tyrosine-derived carbon flux limited performance and 60 mg/L rosmarinic acid was produced. To address this limitation, further pathway partitioning into three strains, two upstream strains producing caffeic acid and salvianic acid A separately and one downstream strain synthesizing rosmarinic acid (Figure 4), combined with the use of mixed carbon substrates, reduced the competition for upstream carbon flux and resulted in 172 mg/L rosmarinic acid production where a 38-fold increase was observed relative to the monoculture (4.5 mg/L) (Li et al., 2019).

**FIGURE 5:**
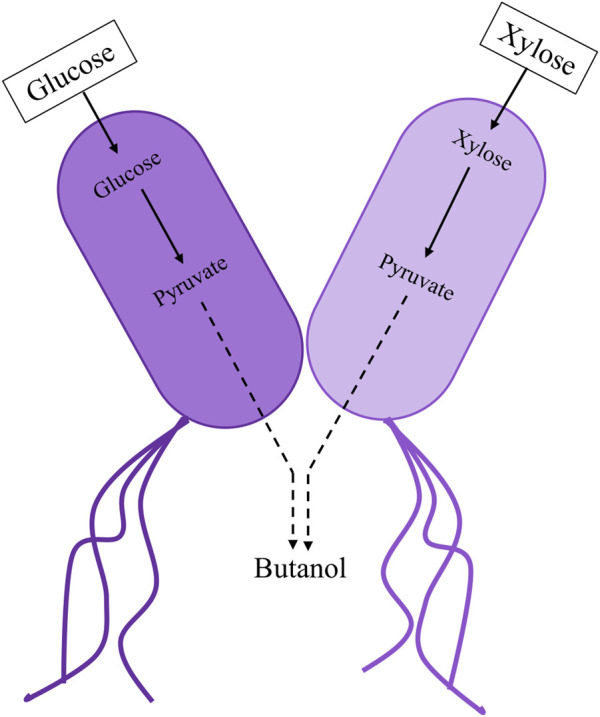
Y-shaped production (type H/H) of butanol with helpful contributions of both *E. coli* partners of the binary consortium (Zhao et al., 2019). While survival of both partners does not depend on the other (0/0), both add to butanol production (H/H), they differ by the carbon and energy source utilized (either xylose or glucose) (Table 3).

The division of labor strategy can also be applied to the use of substrate by the members of the consortium. Microbial production of chemicals from lignocellulosic biomass is limited by the inefficient co-utilization of C5 and C6 sugars. In natural ecosystems, this limitation is mitigated by the cooperation of microorganisms that specialize in different sugars. However, the metabolic diversity of native microbes makes it challenging to coordinate sugar consumption toward a targeted product. To address this, a “Y-shaped” consortium comprising two *E. coli* strains was developed to simultaneously and efficiently utilize mixed sugars (Figure 5). Specifically, both *E. coli* strains shared the same pathway from pyruvate to n-butanol but used different utilization pathways for glucose and xylose. This Y-shaped consortium achieved efficient butanol production from hydrolysates through carbon source partitioning (Zhao et al., 2019).

**FIGURE 6:**
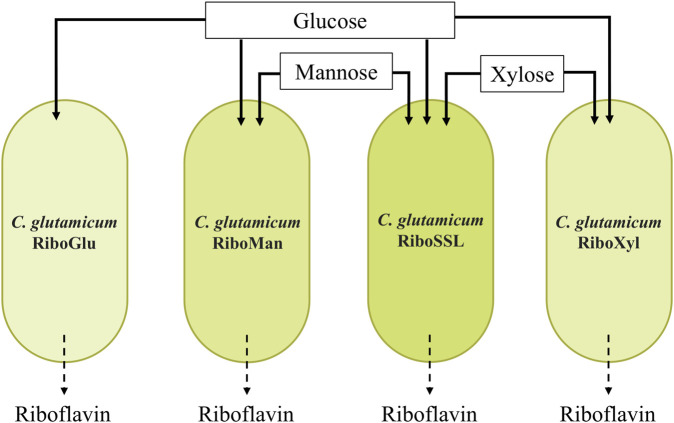
Tooth squeege-shaped production (type H/H/H/H) of riboflavin by a quaternary *C. glutamicum* consortium (Pérez-García et al., 2021). All partners help to produce riboflavin. They differ by their capacities to utilize glucose, mannose, and xylose as carbon and energy sources (Table 1).

Spent sulfite liquors (SSLs) are the main by-product of the sulfite pulping process, with a global annual production of approximately 1.8 million tons (Aro and Fatehi, 2017). SSLs contain high levels of fermentable sugars, predominantly glucose, xylose, and mannose. Riboflavin production from SSL was evaluated using consortia of *C. glutamicum* strains capable of utilizing glucose alone (RiboGlu), glucose plus mannose (RiboMan), glucose plus xylose (RiboXyl), or all three sugars (RiboSSL) (Figure 6). Mannose metabolism was enhanced by overexpressing the native *manA* gene, while xylose consumption was enabled by heterologous expression of *xylA* from *Xanthomonas campestris* together with endogenous *xylB*. Riboflavin production was enabled by overexpression of the *sigH* gene from *C. glutamicum*. The strains were evaluated as monocultures in batch fermentations. As their production capacities were different on different carbon sources and on synthetic SSL medium, RiboMan and RiboXyl were chosen for the dynamic co-cultivation strategy and sugar concentrations were quantified every 2 h. When a sugar was no longer utilized or was utilized more slowly, the respective strain able to utilize it was added. Within the synthetic SSL medium, a 56% higher volumetric productivity combined with 45% less by-product formation compared with an equivalent process inoculated with a single strain (RiboSSL) overexpressing both *xylAB* and *manA* was observed (Pérez-García et al., 2021).

## 3 Production by microbial consortia stabilized by growth dependencies

The long-term stability of synthetic microbial consortia is critical in bioprocesses, since interactions among consortium members strongly influence both system stability and overall performance and, therefore, are under intense evolutionary pressure. In a division of labor binary production consortium, it is likely that the faster-growing partner will completely take over the cultivation. By contrast, when members of a microbial consortium are interdependent, more stable interactions result. Consequently, stable synthetic microbial consortia have to be designed such that interdependencies support sustained cell growth while reliably carrying out their intended producing functions (Jiang et al., 2023).

Guided by naturally occurring stable microbial consortia, growth dependency strategies fall into different categories: (i) auxotrophy complementation *-* one partner supplies essential metabolites to support the growth of the other partner; (ii) substrate facilitation - one partner provides various hydrolases to produce free sugars for the growth of other partner; and (iii) inhibitor elimination *-* one partner removes inhibitory compounds produced by the other and provides a favorable environment for partner growth. These mechanisms differ in strength and impact on consortium stability and should be chosen based on production and process requirements. Auxotrophy complementation enforces obligatory commensalism/mutualism through essential metabolite exchange, resulting in high stability but limited flexibility and potential productivity constraints. Substrate facilitation enables division of labor *via* extracellular substrate breakdown, improving resource utilization but providing moderate stability due to risks of imbalance. Elimination of inhibitors enhances process robustness by removing inhibitory compounds and provides conditional stability as dependency is based on the inhibitor concentration (Bhatia et al., 2018; Jiang et al., 2023).

**FIGURE 7:**
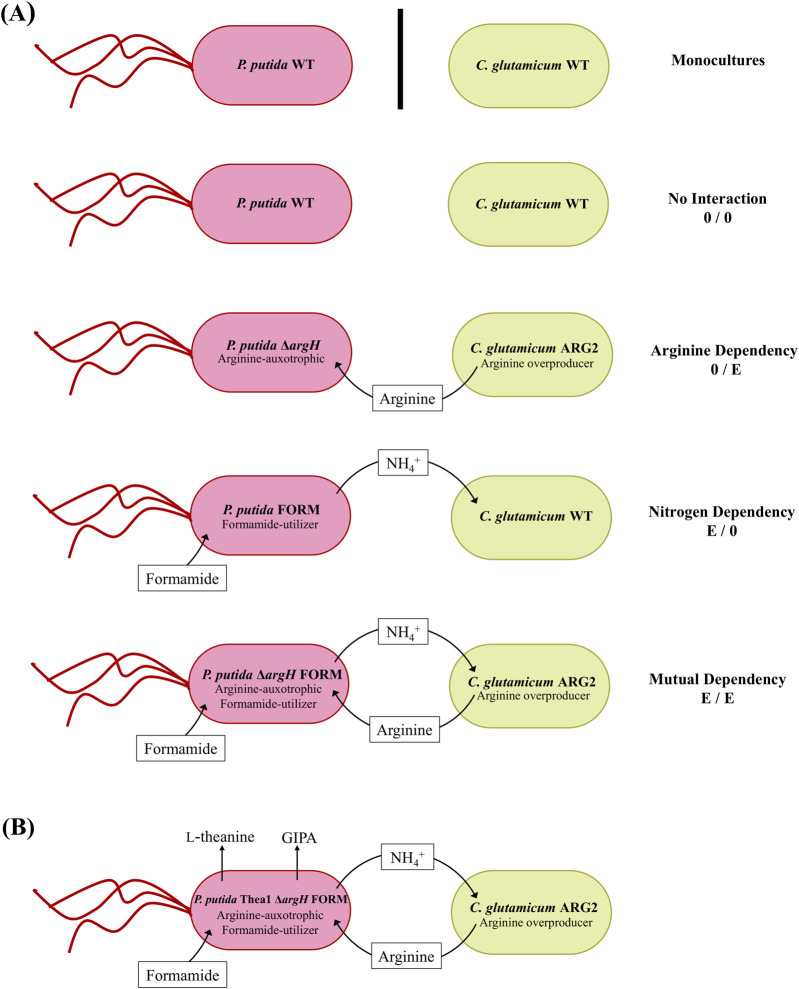
Stepwise design **(A)** of a binary mutualistic (type E/E) *P. putida*–*C. glutamicum* consortium for **(B)** production (type R/N) of the amino acids L-theanine or GIPA (Benninghaus et al., 2024). **(A)** Based on the ability to utilize formamide as a sole nitrogen source (or not) and to complement an arginine auxotrophy by arginine overproduction (or not), a binary mutualistic consortium (type E/E) was designed and gradually implemented starting from monocultures *via* stages of neutralism and commensalism. **(B)** In the presence of ethylamine or isopropylamine, the *P. putida* recombinant produces L-theanine or GIPA, respectively. The *C. glutamicum* recombinant did not directly contribute to production (R/N). The *P. putida* recombinant only grows if its arginine auxotrophy is complemented by an arginine overproducing *C. glutamicum* recombinant, which in turn only grows upon provision of ammonia from the formamidase-positive *P. putida* strain. In a medium containing arginine, the *P. putida* recombinant is able to produce L-theanine or GIPA independently of the *C. glutamicum* strain (Table 1).

Different dependency strategies have been used to construct binary, ternary, and quaternary microbial consortia (Tables 1–3). A stepwise approach was applied to develop a binary consortium of *P. putida–C. glutamicum*, resulting in a mutualistic system capable of producing L-theanine or GIPA (Benninghaus et al., 2024). Initially, both strains were co-cultivated in a shared medium that supported the independent growth of each organism. In this case, neither strain depended on the other, corresponding to a neutral interaction (0/0; neutralism). Next, two commensal consortia were established, where one partner benefited while the other was unaffected. In the first case, an arginine-auxotrophic *P. putida* strain depended on an arginine-overproducing *C. glutamicum* strain, while *C. glutamicum* remained unaffected (type E/0). In the second case, a formamidase-positive *P. putida* strain supplied ammonium from formamide, enabling the growth of *C. glutamicum* without receiving a benefit in return (type 0/E). Finally, these dependencies were combined to create a mutualistic consortium (type E/E), in which both partners relied on each other. The engineered *P. putida* strain required arginine from *C. glutamicum*, while *C. glutamicum* depended on *P. putida* for ammonium supply. Production of L-theanine or GIPA was achieved by introducing enzymes for γ-glutamylation of ethylamine or isopropylamine, respectively, into the engineered *P. putida* strain (Figure 7) (Benninghaus et al., 2024).

**FIGURE 8:**
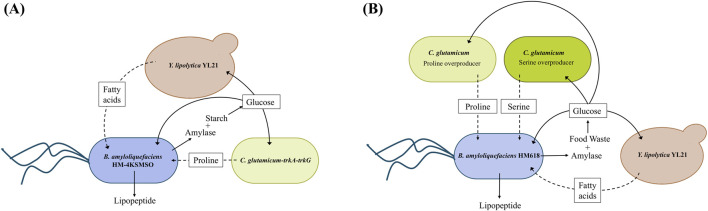
Production of lipopeptide from starch (A; type R/H/H) by a ternary commensal consortium (Zhang et al., 2024) and from food waste (B; type R/H/H/H) by a quaternary neutralistic consortium (He et al., 2024). **(A)** On its own, the *B. amyloliquefaciens* strain is able to produce lipopetides from starch. By providing proline and fatty acids, respectively, as precursors, both the *Y. lipolytica* strain and the *C. glutamicum* strain help lipopeptide production (type R/H/H), but in this commensal consortium (0/E/E), they can only grow with starch when amylase is secreted by the *B. amyloliquefaciens* strain. **(B)** The concept was extended by an additional *C. glutamicum* strain producing serine as precursor for lipopeptide production (type R/H/H/H). Commensal consortium produces lipopeptide from starch in food waste (0/E/E/E) (Table 1).

While in the example above, complementation of an amino acid auxotrophy was combined with access to a nitrogen source (here, the rare formamide), amino acid auxotrophies were combined with access to a carbon source. For example, a lysine-overproducing *C. glutamicum* strain could only access starch or chitin when co-cultured with a lysine-auxotrophic *E. coli* strain that secreted either starch- or chitin-degrading enzymes (Sgobba et al., 2018; Vortmann et al., 2021). In other examples, a *B. amyloliquefaciens* amylase-secreting strain degraded starch and, thus, enabled access to the carbon source glucose for the other microbial partners of a ternary and a quaternary consortium, respectively, that are unable to degrade starch (He et al., 2024; Zhang et al., 2024). Specifically, in the ternary consortium for lipopeptide production by *B. amyloliquefaciens* from starch (0/E/E; Figure 8A), *C. glutamicum* and *Y. lipolytica* relied on amylase secreted by *B. amyloliquefaciens* for access to glucose from starch. The *C. glutamicum* and *Y. lipolytica* strains enhanced lipopeptide synthesis by providing proline and fatty acids (R/H/H) (Zhang et al., 2024). With the addition of a serine-overproducing *C. glutamicum* strain, the system was extended to a quaternary consortium (Figure 8B). Through the degredation of starch-containing food waste into glucose as a carbon substrate *via* the amylase-secreting *B. amyloliquefaciens* strain (0/E/E/E), lipopeptide production by this strain benefited from serine, proline, and fatty acids as lipopeptide precursors, which were provided by the other three partner strains (R/H/H/H) (He et al., 2024).

## 4 Stabilization of microbial consortia by detoxification of inhibitory compounds

Elimination of toxic compounds or by-products can also enhance the stability of the consortia (Jiang et al., 2023). Although the use of lignocellulosic feedstocks as a carbon source offers a sustainable solution to bioproduction, their use is constrained by toxic by-products generated during pretreatment, which impair microbial growth and productivity. For example, hydrolysis of hemicellulosics by dilute sulfuric acid typically yields xylose, glucose, and arabinose as carbon sources, and at about tenfold lower concentrations of the growth inhibitory compounds acetic acid, phenolic compounds, 5-hydroxymethylfurfural, furfural, and formic acid (Rama et al., 2025). Production of 2,3-butanediol from hemicellulosic hydrolysates was enhanced when the *P. polymyxa* producer strain was used in a binary consortium with *P. putida,* as it degrades furans and phenolic compounds (Rama et al., 2025). The same concept, i.e., co-culturing with *P. putida*, improved hemicellulosic hydrolysate-based L-lactate production with *B. coagulans* (Zou et al., 2021).

Growth inhibitory compounds do not only arise from chemical treatments such as acid hydrolysis of lignocellulosic material, but are generated in cellular metabolism, as well. Acetic acid is secreted during aerobic, incomplete carbohydrate oxidation or during a number of anaerobic fermentation processes. Growth of *E. limosum* with carbon monoxide (CO) yields acetate as a product. However, *E. limosum* cultures suffer from the accumulation of acetic acid (Cha et al., 2021). Co-culturing an *E. coli* strain with *E. limosum* overcame this growth inhibition since *E. coli* utilized the acetic acid formed by *E. limosum.* Using engineered *E. coli* strains in the consortium with *E. limosum* not only alleviated the growth inhibition of *E. limosum* by acetic acid, but also allowed for acetate-based production of itaconic acid and 3-hydroxypropionic acid (Cha et al., 2021). Thus, in this approach, acetic acid was converted from a growth-inhibitory product to a substrate for the production of itaconic acid or 3-hydroxypropionic acid.

## 5 Adaptive laboratory evolution to improve strains for use in microbial consortia

Adaptive laboratory evolution (ALE) is a powerful strategy for generating microorganisms with enhanced functional traits. In monocultures, ALE has been widely applied to improve growth, stress tolerance, substrate utilization, and production performance. Extending ALE to synthetic microbial consortia offers opportunities to enhance community stability, metabolic cooperation, and overall functional performance, while also providing insights into the evolutionary dynamics of engineered ecosystems (Zhang and Reed, 2014; Wen et al., 2020; Konstantinidis et al., 2021).

ALE has also been applied to improve one partner for subsequent use in a consortium. For example, ALE was applied to enhance α-pinene tolerance and production in an *E. coli* strain (Niu et al., 2018). Following atmospheric and room temperature plasma mutagenesis of an engineered strain harboring a heterologous pinene biosynthetic pathway was serially passaged under increasing pinene concentrations. Of the selected mutants that grew in the presence of 2.0% pinene, one isolate showed increased pinene production and was subsequently incorporated into a microbial consortium with a partner strain secreting isopentenyl pyrophosphate (Niu et al., 2018). In a similar application, ALE improved the acetate tolerance of an *E. coli* strain that efficiently utilized acetate as it lacked four genes (*adhE*, *pta*, *ldhA*, and *frdA*) involved in acetyl-CoA consumption. In co-culture with its autotrophic partner *M. capsulatus* Bath, this evolved strain supported more efficient conversion of methane-derived organic acids into mevalonate through an engineered heterologous pathway (Seong et al., 2020). Other examples have been described, e.g., to improve tolerance to salt when using food waste and seawater for lipopeptide production (Zhang et al., 2024) or capsaicinoid-rich food waste for fengycin production (Duan et al., 2025). These examples are helpful to improve one partner for subsequent use in a consortium, but these approaches cannot drive emergent system properties.

## 6 Adaptive laboratory evolution to improve production by microbial consortia stabilized by growth dependencies

ALE has been used to improve performance on synthetic microbial consortia. Growth-coupled pathway engineering combined with flux balance analysis (FBA) and ALE was applied to construct an *E. coli–E. coli* consortium for phenylalanine production. FBA predicted a set of gene deletions (*pykA*, *pykF*, *ppc*, *zwf*, and *adhE*) to couple phenylalanine biosynthesis with growth, but the resulting engineered strain (KF) exhibited severe growth defects. To address the absence of selective pressure for phenylalanine production under low growth conditions, a mutualistic co-culture system involving two distinct auxotrophs, one producing phenylalanine and auxotrophic for leucine (KF), and the other producing leucine and auxotrophic for phenylalanine, was employed, enabling the coupling of growth and production independently of growth yield. After 160 generations, an evolved strain (KF-E) was obtained from the culture exhibiting the highest specific growth rate and this KF-E strain produced phenylalanine at a yield 2.3 times greater than that of the KF strain (Kawai et al., 2022). The ability of the KF-E strain to grow in monoculture represents a significant advancement toward further improving productivity.

## 7 Metabolic costs of consortia: export and import of intermediates

**FIGURE 9:**
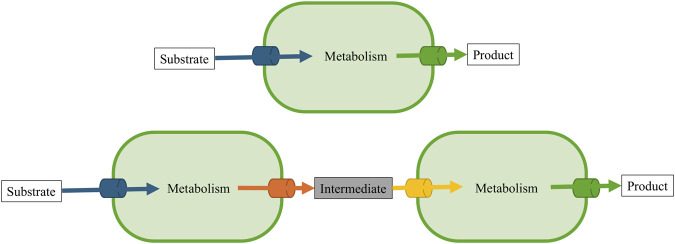
Involvement of transport systems for production by a monoculture as compared to a consortium. Uptake of the substrate (blue), secretion (orange), uptake of the intermediate secretion (yellow), and secretion of the product (green) are indicated.

The import of substrates and export of final products are critical determinants of production by fermentation. Transport proteins play a central role in enabling efficient metabolite flux across cellular membranes. In strain development, transport engineering aims at improving product export and substrate uptake and/or avoiding loss of intermediates by excretion (Pérez-García and Wendisch, 2018). Transport is even more relevant in microbial consortia (Figure 9), where the transport of pathway intermediates between different microbial partners is essential for division of labor strategies and overall system performance (Sgobba and Wendisch, 2020; Yamada et al., 2024). In other words, division of labor between several partners of a consortium comes at a cost: excretion of an intermediate by one partner and uptake of it by the other may require ATP, as for primary active transport systems or gradients of ions or co-substrates as for secondary active transport systems. Metabolite transport can become problematic when a pathway is split between hosts, requiring the intermediate product to move from one organism to another for further conversion. If this intermediate is unstable, unable to cross the cell membrane, or if the receiving host lacks the appropriate transporter to import it, the division of labor will fail. Moreover, designing efficient transporters and efflux systems is challenging and can impose additional metabolic burdens (Tsoi et al., 2018; Roell et al., 2019). Because the host may consume additional ATP/NADH, and in general, it is estimated that up to 60% of an organism’s total ATP demand is spent on the activity of its transportome (Darbani et al., 2018). Thus, the advantage of division of labor regarding product formation must be higher than the transport costs imposed in synthetic microbial consortia.

Substrate uptake and product export are, of course, regular metabolic engineering targets when designing microbial consortia. Export of end products was engineered by expression of the *E. coli* AcrAB pump and the *P. putida* TtgB pump genes and shown to improve both pinene tolerance and its production in a co-culture system (Dunlop et al., 2011; Niu et al., 2018). Engineering export systems was also shown to be relevant in the co-cultivation of photosynthetic microorganisms with heterotrophic bacteria that have recently attracted considerable attention as a promising platform for sustainable bioproduction (Scognamiglio et al., 2021). In such systems, the phototrophic partner fixes CO_2_ and supplies organic carbon compounds, while the heterotrophic partner consumes these compounds and releases CO_2_ through respiration, thereby supporting phototrophic growth. *S. elongatus* has been engineered to secrete sucrose *via* chromosomal integration of the *cscB* gene, encoding a sucrose/H^+^ symporter from *E. coli* (Ducat et al., 2012). Phototrophic-heterotrophic consortia with this engineered cyanobacterium have been widely employed, e.g., with *C. glutamicum* for *cis,cis*-muconate production (Ma et al., 2026) and with *P. putida* for the biotransformation of 5-hydroxymethylfurfural to 2,5-furandicarboxylic acid as well as for the production of polyhydroxyalkanoate (Hobmeier et al., 2020; Lin et al., 2020; Kratzl et al., 2023). Glycerol uptake has been engineered in a *C. glutamicum* strain by heterologous expression of the glycerol facilitator gene *glpF* along with the glycerol utilization genes *dhaDK* for application in a *C. glutamicum*–*E. coli* consortium producing cadaverine from glycerol (Liu S. et al., 2022). Besides substrate uptake, the *E. coli* strain of this consortium was also engineered for improved uptake of the intermediate lysine and export of the product cadaverine by introducing the lysine–cadaverine antiporter CadB (Liu S. et al., 2022). While in this consortium export of the intermediate was not engineered, a number of examples are known in which an amino acid is exported as an intermediate for further conversion by the microbial partner, and therefore, amino acid export has been engineered (See below).

Gargatte et al. constructed an *E. coli*–*E. coli* co-culture system for 4-hydroxystyrene production *via* tyrosine. An aromatic amino acid exporter, PhpCAT from *Petunia hybrida*, was expressed to enhance tyrosine secretion, resulting in a 96% increase in 4-hydroxystyrene production compared to the control system (Gargatte et al., 2021). Similarly, a synthetic consortium of *E. coli* and *M. guilliermondii* was developed for 2-phenylethanol production, where overexpression of the phenylalanine permease, GAP, in *M. guilliermondii* significantly improved product titers (Yan et al., 2022). Furthermore, fengycin production was enhanced in a binary consortium with a proline-overproducing *C. glutamicum* strain through the expression of the proline transporter, OpuE, in *B. subtilis* (Gao et al., 2023). Overexpression of *opuE* also benefited lipopeptide production by providing proline as an intermediate in ternary consortia involving *B. amyloliquefaciens*, *C. glutamicum*, and *Y. lipolytica* (Zhang et al., 2024). Overexpression of the gene encoding the L-serine exporter SerE from *C. glutamicum,* in addition to *opuE* improved the provision of serine and proline for lipopeptide production in a quaternary consortium (Sun et al., 2022).

The amino acids phenylalanine and tyrosine were key intermediates in the production of D-*p*-hydroxyphenylglycine from glucose by a mutualistic consortium composed of a phenylalanine-auxotrophic *P. putida* strain and a tyrosine-auxotrophic *E. coli* strain. First, these amino acids served as metabolic intermediates, enabling the division of labor between the two strains. Phenylalanine produced by *E. coli* was supplied to *P. putida*, which converts it to D-*p*-hydroxyphenylglycine *via* tyrosine. To improve this intermediate exchange, amino acid transport was engineered in both partners. Overexpression of the aromatic amino acid exporter gene *yddG* in phenylalanine-producing *E. coli* enhanced phenylalanine secretion, while expression of the *E. coli* L-phenylalanine permease PheP in *P. putida* improved its uptake into the *P. putida* cell (Liu et al., 2022e). Similarly, tyrosine transport was optimized to support pathway flux. Tyrosine synthesized by the *P. putida* strain was sufficient to complement the tyrosine-auxotrophic *E. coli* strain, which overexpressed endogenous *tyrP* to improve tyrosine uptake. Second, phenylalanine and tyrosine exchange also stabilized the consortium through auxotrophy complementation. The phenylalanine-auxotrophic *P. putida* depended on *E. coli* for phenylalanine, while the tyrosine-auxotrophic *E. coli* relied on *P. putida* for tyrosine. This example demonstrated how transport engineering can simultaneously enhance pathway efficiency (by improving intermediate exchange) and enforce population stability (through auxotrophy complementation). In contrast to other systems where these functions are addressed separately, here both objectives are achieved through the coordinated optimization of the same metabolite exchange processes.

Transporters also contribute to microbial tolerance against toxic compounds. In a synthetic consortium of *B. subtilis*, *C. glutamicum*, and *Y. lipolytica* producing fengycin from capsaicinoid-rich kitchen waste, overexpression of the transporters YtrBCDEF and LmrB enhanced capsaicinoid tolerance in *B. subtilis*, indirectly benefiting fengycin production (Duan et al., 2025).

Taken together, transport engineering emerges as a key strategy for optimizing substrate uptake, precursor supply, intermediate exchange, and product secretion in microbial consortia. Developing elaborated strategies for transport engineering, including metabolite-responsive, biosensor-controlled expression of uptake and/or export genes, will be essential for constructing robust, high-performance synthetic consortia for sustainable bioproduction.

## 8 Combining production by microbial consortia and follow-up chemistry in one-pot formats

The principle of division of labor within microbial consortia can be extended to follow-up chemistry, i.e., a chemical reaction converts the compound synthesized by the microbial consortia to the final product. In biocatalysis, this principle has been used to convert the compound synthesized by an enzyme or enzyme cascade *via* a subsequent chemical reaction (Buller et al., 2023). Moreover, in a reverse sequence, a compound synthesized through a chemical reaction was subsequently converted by an enzyme or enzyme cascade to the final product (Gröger et al., 2022). Thus, we propose that future synthesis concepts may cascade reactions catalyzed either by microbial strains, enzymes, or synthetic chemistry.

The structurally complex pyranoanthocyanins that are naturally formed during the fermentation and aging of red wine exhibit strong antioxidant activity and contribute natural coloration to foods and beverages. Since their isolation is particularly challenging due to their low natural concentrations (Topić Božič et al., 2020), binary co-culture systems using recombinant *E. coli* strains have been developed. In one system, *E. coli* strains were engineered to produce 4-vinylphenol and cyanidin-3-*O*-glucoside, and pyranocyanidin-3-*O*-glucoside-phenol is formed by direct chemical reaction from these precursors. Similarly, another co-culture system utilized strains producing 4-vinylcatechol and cyanidin-3-*O*-glucoside, respectively, which react to pyranocyanidin-3-*O*-glucoside-catechol. Upon optimization of inoculum ratio and induction timing combined with mimicking a wine-making environment, maximum titers of 19.5 mg/L for pyranocyanidin-3-*O*-glucoside-phenol and 13.2 mg/L for pyranocyanidin-3-*O*-glucoside-catechol were obtained. This strategy enabled the production of pyranoanthocyanins with greater stability compared to conventional extraction methods from plants (Akdemir et al., 2019).

Dying chemistry was combined with fermentative production of bacterial cellulose. As opposed to reactive dying or VAT dying, direct dying of bacterial cellulose by a consortium of a cellulose-overproducing *K. xylinus* strain and a colorant-producing *E. coli* strain was achieved. The *E. coli* strain either secreted proviolacein (green), prodeoxyviolacein (blue), violacein (navy) or deoxyviolacein (purple) or accumulated carotenoids astaxanthin (red), *β*-carotene (orange) or zeaxanthin (yellow) in its membranes. Vesicle and membrane engineering reduced cytotoxicity and facilitated more efficient pigment secretion. In the delayed co-culture approach, the violacein derivative-secreting *E. coli* strain was added to the flasks after the *K. xylinus* strain produced the bacterial cellulose. Thus, dying occurred in a one-pot system with both bacteria growing and producing in the same flask. Remarkably, the bacterial cellulose colored by secreted violacein derivatives showed comparably stable retention of these colors even after numerous washing cycles and high-temperature drying. In the future, other color-producing strains can be used to dye bacterial cellulose produced by the *K. xylinus* strain (Zhou et al., 2025).

## 9 Discussion

Microbial consortia occupy important habitats and niches in nature. In recent years, biotechnology has witnessed the development of the concept of synthetic microbial consortia as well as the first applications to bioproduction. This trend is on the rise with about 1000 PubMed entries for “synthetic microbial consortia”, more than 20% have been published in the last year, 2025. Many consortia have been designed and implemented to demonstrate that microbial cooperation can be realized with the purpose of bioproduction in mind.

The current limitations are not due to technological boundaries, but rather to make sure that the advantage of dividing a task, such as fermentative production of a chemical compound between microbial partners, surpasses the implied metabolic costs, such as for secretion and uptake of intermediates. Stability of the synthetic microbial consortia over (the whole process) time plays an important role. The implementation of interdependencies for stabilization of consortia comes with the burden of a metabolic cost, as well. Most interdependencies realized are based on auxotrophies. Thus, they are inherently growth-associated, which limits their relevance for control of the production of specialized metabolic products once growth has ceased. In more general terms, the design aim regarding synthetic microbial consortia typically is to fix the composition of the sub-populations at a pre-defined ratio, while temporal patterning may be beneficial in certain applications. The scientific community has yet to develop metrics that provide quantitative measures guiding the design and realization of microbial consortia.

Quantifying the performance of consortia relative to monocultures is critical for industrial relevance. For instance, in rosmarinic acid production, partitioning the pathway into a three-strain consortium increased production ∼38 fold relative to the monoculture (Li et al., 2019). Similarly, dynamic co-cultivation strategies in lignocellulosic sugar utilization increased volumetric productivity of riboflavin by 56% and reduced byproduct formation by 45% compared to a single strain harboring all metabolic functions (Pérez-García et al., 2021). These data indicate that consortia can surpass monoculture performance when metabolic burden or substrate utilization limitations constrain single hosts. Together, these quantitative improvements demonstrate that microbial consortia are not only conceptually advantageous but also have practical potential for industrial-scale applications.

Nature provides examples, as does the fermentation industry, e.g., when considering the successions of salt-tolerant yeasts and bacteria of the phyla Actinobacteria, Firmicutes, and Proteobacteria involved in ripening of red-orange surface smear cheeses (Ritschard and Schuppler, 2024). The application of microbial consortia in industry is well established. Microbial consortia have long been used in the commercial production of fermented foods such as vinegar, soy sauce, and cheese. In addition, consortia-driven processes play key roles in municipal and industrial wastewater treatment, biogas production, and environmental remediation, and are also applied in the mining industry for mineral extraction from ores (Sabra et al., 2010; Bernstein and Carlson, 2012). Despite these applications, industrial use of microbial consortia presents several challenges, including maintaining stable microbial communities, improving performance, and scaling processes from laboratory to industrial settings. The complex interactions within these systems make them harder to control, predict, and monitor in real time (Kumar et al., 2025).

Ecological niches typically present spatial patterning and (step) gradients in physical parameters, such as pH, liquid and gas exchange, or mechanical obstacles imposed by pore sizes, which influence the distribution of the cooperating microbial partners present. Compartmentalization applied to a synthetic microbial consortium has been described, e.g., for the production of indigoidine in a photo-bioreactor by a microbial consortium with phototrophic *S. elongatus* and indigoidine-producing *P. putida* encapsulated in calcium-alginate hydrogel beads (Zhao et al., 2022). However, the concept of spatial niche patterning is only in its infancy.

Given the momentum of intensifying research on synthetic microbial consortia, we forecast many as well as groundbreaking developments to design efficient, time- and space-controlled consortia for application in bioproduction of valuable compounds. The technological toolbox is versatile and adequate to address these challenges already now; however, the foreseeable technological improvements in genetics, biochemistry, and physiology yet to come will for sure find their way into the engineering of synthetic microbial consortia.
